# Disparity in prevalence and predictors of undernutrition in children under five among agricultural, pastoral, and agro-pastoral ecological zones of Karamoja sub-region, Uganda: a cross sectional study

**DOI:** 10.1186/s12887-022-03363-6

**Published:** 2022-05-30

**Authors:** Lawrence Okidi, Duncan Ongeng, Patrick Simiyu Muliro, Joseph Wafula Matofari

**Affiliations:** 1 Department of Dairy and Food Science and Technology, Egerton University, P.O.Box 536 - 20115, Egerton-Njoro, Kenya; 2 Department of Food Science and Postharvest Technology, Gulu University, P.O.Box 166, Gulu, Uganda

**Keywords:** Ecology, Food handling, Undernutrition, Children under five

## Abstract

**Background:**

Undernutrition accounts for nearly half of under-five child mortality in developing countries where household nutrition is largely dependent on agriculture. Despite the strong influence of agroecology on agriculture in those countries, limited information exists on whether undernutrition in children under five varies with agro-ecological location.

**Methods:**

Using Karamoja sub-region of Uganda, one of the most food insecure parts of Eastern Africa as a case area, and applying a multi-stage sampling procedure, and a structured questionnaire, this study examined in a comparative manner, the prevalence and predictors of undernutrition in children under five among the agricultural, pastoral, and agro-pastoral ecological zones. Chi-square test and Kruskal-Wallis test were used to establish the disparity in prevalence of undernutrition and household contextual characteristics, respectively. Binary logistic regression was used to determine the predictors of undernutrition in children under five among the three agro-ecological zones. The level of statistical significance was set at *p* ≤ 0.05.

**Results:**

The prevalence of underweight, stunting, and wasting ranged from 36 to 58% but varied with agroecology in terms of the peak age ranging from 6 to 37 months. Child characteristics, feeding practices, household economic factors, sanitation factors, and caregiver characteristics that predict undernutrition among children under five were identified (*p* ≤ 0.05). Caregiver handwashing after using latrine (*p* = 0.005) and diarrhoea in a fortnight (*p* < 0.001) increased the likelihood of stunting in pastoral agroecology only whereas cereal storage in both sacks and granary in agro-pastoral zone was associated with reduced likelihood of both underweight (*p* < 0.001 and *p* = 0.014) and stunting (*p* = 0.011 and *p* = 0.018), respectively. A male child was more likely to be underweight and stunted in pastoral (*p* = 0.002 and *p* = 0.011) and agro-pastoral (*p* = 0.017 and *p* = 0.002) agroecology, respectively. Household expenses reduced the likelihood of both underweight and wasting in pastoral (*p* = 0.013 and *p* = 0.005) and agricultural (*p* = 0.011 and *p* = 0.021) agroecology, respectively. Flour storage duration increased the stunting likelihood in pastoral (*p* = 0.032) and agro-pastoral (*p* = 0.006) agroecologies.

**Conclusion:**

This study has revealed that, in a food insecure developing country setting such as Karamoja sub-region of Uganda, undernutrition among children under five varies with agroecology. Thus, nutritional interventions in such locations should be agroecology specific.

**Supplementary Information:**

The online version contains supplementary material available at 10.1186/s12887-022-03363-6.

## Background

Undernutrition is still one of the leading public health challenges in many developing countries majorly in Southeast Asia and Sub-Saharan Africa (SSA) [1]. Undernutrition accounts for nearly 50% of all mortalities of children under five globally [2], and largely affects this age category due to their increased nutrient requirement for rapid physical, immunological, cognitive growth, and development [3]. Among this age group, undernutrition is often exhibited as low weight for age (underweight), low height for age (stunting), and low weight for height (wasting) [4–6]. These anthropometric indices are considered to be low when they fall two standard deviations below the National Centre for Health Statistics (NCHS)/ World Health Organisation (WHO) median reference value for a population [7]. Globally, it is estimated that 21.3% (144 million) and 6.9% (47 million) of children under five are stunted and wasted, respectively [2]. In SSA, the level of underweight, stunting, and wasting stands at 21, 32, and 7% [8, 9] while for Uganda, it stands at 11, 29, and 4%, respectively. In Uganda, Karamoja sub-region, one of the most food insecure locations in Eastern Africa has the highest level of child undernutrition estimated at 26, 35, and 10%, for underweight, stunting, and wasting, respectively [10]. Indeed, food insecurity is a known risk factor for undernutrition [11, 12]. As such, these levels of child undernutrition indicators are considered to be high based on WHO cut-off of < 10, < 20, and < 5% for tolerable level of undernutrition in a given community for underweight, stunting, and wasting, respectively [13].

Undernutrition in children under five presents both short and long-term developmental effects. Short-term effects on child health include; compromised immunity, reduced growth rate which leads to increased susceptibility to diseases associated with increased morbidity and mortality whereas long-term developmental effects include compromised brain development which limits attainment of full potential at adulthood [14]. Owing to the short and long-term consequences of undernutrition in children under five, addressing undernutrition requires development of strategies that addresses the underlying causes in a given societal context [15]. Traditionally in the African set-up, in pastoral areas such as Karamoja sub-region of Uganda, communities were known to be reliant on animal-based livelihoods and movement from place to place [16]. However, over the years, the inhabitants of the sub-region have evolved from purely pastoralist livelihood to agro-pastoralists, pastoralists, and agriculturalists [17]. This has consequently resulted into changing livelihoods from purely animal-based (pastoral) to a mix of both animal and crop-based (agro-pastoral) and purely crop-based (agricultural) [18, 19]. The change in livelihood strategy has consequences for food and nutrition security. For instance, the Maasai community (a pastoral tribe in Kenya and Tanzania) that rely primarily on livestock herding were found to be more food insecure compared to those that depend primarily on agriculture [20]. This suggest that agroecology influences nutrition outcomes among vulnerable segments of the human population such as children under five. However, limited information exists on whether nutrition outcomes among children under five in a food insecure locality such as Karamoja sub-region varies with agro-ecological location. This has implication for tailoring nutritional intervention. Therefore, using Karamoja sub-region of Uganda as a case area for a food insecure location in developing countries in SSA where household nutrition largely depends on agriculture, this study examined whether the prevalence of undernutrition among children under five and associated factors vary with agroecology.

## Methods

### Study design and setting

**Fig. 1:**
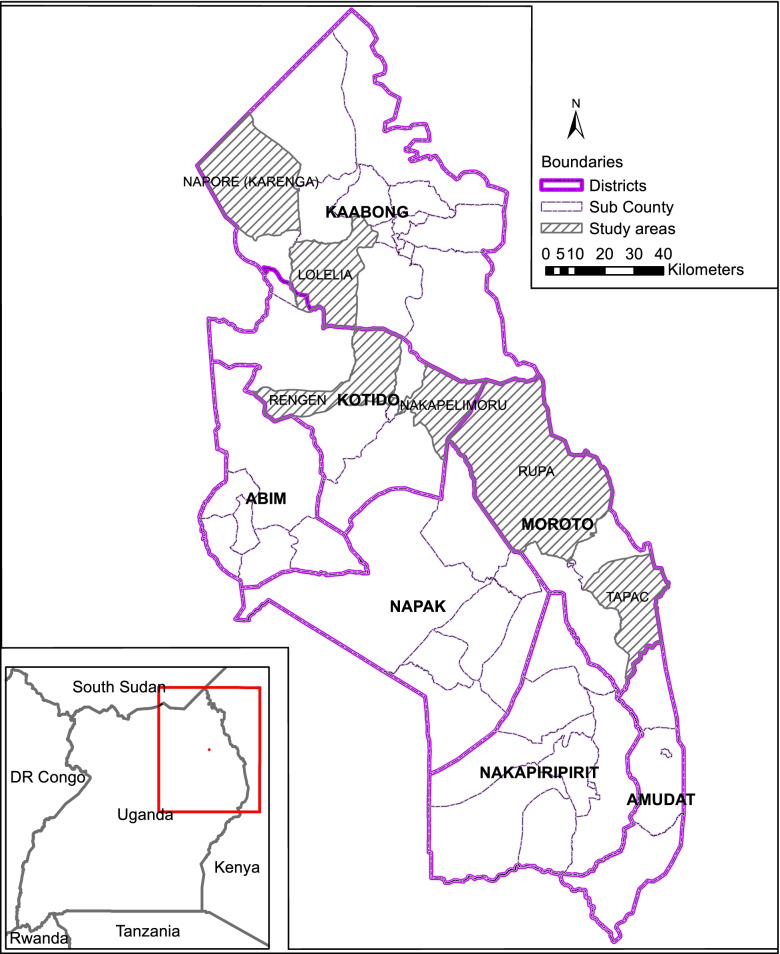
Map showing study area

The study employed a cross-sectional design and was conducted in pastoral, agro-pastoral, and agricultural agro-ecological zones of Karamoja sub-region located in north Eastern Uganda between October and December 2019. The pastoral is a semi-arid zone where livelihoods mainly depend on livestock production (cattle, goats and sheep) with crop cultivation in years of adequate rainfall focused on millet, cowpeas and groundnuts. Average annual rainfall is less than 300–500 mm. Soils are predominantly sandy and of low fertility with Moroto being the only district this zone [21]. The agro-pastoral zone on the other hand receives annual rainfall of 500–800 mm, with rains erratically distributed. The sandy, loamy soils support crops such as sorghum, millet, maize, beans, cowpeas and groundnuts, generally farmed on small plots of land around fenced hamlets, or manyattas, and settlements using intercropping techniques. Livestock production focuses on steers, bulls, sheep and goats. Agro-pastoral zone is constituted by Kotido and Napak districts [21]. The agricultural is a wetter zone of fertile, loamy soils referred to as the “green belt” in the south and west of the region, with average rainfall ranging from 800 to 1200 mm annually and a growing season that extends from March to October. This zone supports a wide variety of crops and can often accommodate a second and third planting of quick-maturing cash and food crops after the maize and bean harvest, such as sesame, sunflower, simsim, cucumber (adekela) and an assortment of local vegetables and fruits (mangoes, oranges, sweet bananas, passion fruit, paw paw). The agricultural zone is consisted of Abim, Nakapiripirit, Amudat, and Kaabong districts [21]. In each agro-ecological zone, a district was purposively selected on the basis of having highest prevalence of Global Acute Malnutrition (GAM). Consequently, Moroto, Kotido, and Kaabong districts were selected to represent pastoral, agro-pastoral, and agricultural agro-ecological zones, respectively. The prevalence of GAM stands at 18.5% in both Kotido and Moroto, whereas that of Kaabong stands at 11.8% [22]. Karamoja sub-region is largely inhabited by the Karamojong and consists of 7 districts (Moroto, Kotido, Napak, Kaabong, Abim, Nakapiripirit, and Amudat). Moroto district is bordered by Kenya to the East, Amudat and Nakapiripirit districts to the South, Napak district to the West, Kotido to the North East, and Kaabong to the North [23]. The main source of livelihood in the district is pastoralism [21]. Kotido district is bordered by Moroto to the East, Napak to the South, Abim district to the West, Kitgum and Agago districts to the North West, and Kaabong to the North [23]. The main source of livelihood in the district is pastoralism and crop agriculture [21]. Kaabong district is bordered by Kotido and Moroto to the South, Kenya to the East, South Sudan to the North, and Kitgum to the West [23]. Livelihoods in Kaabong are majorly reliant on crop agriculture [21]. The population of Moroto, Kotido, and Kaabong stands at 103,432, 181,050, and 167,879 people, respectively. The average household size is 4.4, 6.5, and 5.7 for Moroto, Kotido, and Kaabong districts, respectively [24]. The map of Karamoja sub-region showing the study area is presented in Fig. 1.

### Study population and sampling

The study population were children under 5 years and their mothers/caregivers from three agro-ecological zones of Karamoja sub-region. The number of study participants (sample size, n) was calculated using a standard formula by Kasiulevičius et al. [25] as presented in eq. 1.

1 $$\mathrm{n}=\frac{{\mathrm{z}}^2\ \mathrm{p}\left(1-\mathrm{p}\right)}{{\mathrm{e}}^2}$$

Where; n is the required sample size, Z is the confidence level at 95% (standard value = 1.96), p is the prevalence of GAM among children under five in Karamoja sub-region estimated at 13.8% [22], e is the margin of error set at 5% (standard value =0.05). Using Eq. 1, the minimum sample size was determined to be 183.

Multi-stage sampling procedure was used to draw the study respondents. A total of 55, 96, and 89 households were drawn from the districts that are representative of each agro-ecological zone using probability proportional to size sampling procedure. These were Moroto, Kotido, and Kaabong districts for pastoral, agro-pastoral, and agricultural agro-ecological zones, respectively. This sampling procedure was based on a population of 103,432, 181,050, and 167,879 people in Moroto, Kotido, and Kaabong districts, respectively [24]. On the basis of multi-stage sampling procedure, the sample size was adjusted to 240 households to reduce sampling errors [26]. Prior to random selection of the sub-counties and parishes, a complete enumeration of the sub-counties in each district and parishes in each sub-county were done by the district community development officer and sub-county development officer, respectively. Two sub-counties were randomly selected from each district, followed by random selection of two parishes from each of the selected sub-counties. Finally, using a simple random sampling technique a total of 13–14, 24, and 22–23 households were selected per parish to participate in the study for Moroto (pastoral ecology), Kotido (agro-pastoral ecology), and Kaabong (agricultural ecology) districts, respectively. In a situation where a household selected had more than one child under five (eligible for the study), random selection was used to pick the child for inclusion in the study. Briefly, the age of each child under five eligible for the study in a given household was recorded on the interview questionnaire. The age corresponding to each child was then written on a small piece of paper, folded, and mixed by shaking while covered by both palms. The palms were then opened and the mother or caregiver was asked to pick any of the folded pieces of paper. The child whose age was in the paper selected by the caregiver or mother was then included in the study. Random selection was used to enable all children under five in a given household to have equal chances of participating in the study [27].

### Inclusion criteria

Children aged 6–59 months in selected parishes that had been selected for inclusion in the sample and households having at least one child under the age of five and have been residing in a given parish for more than 1 year were included in the study.

### Exclusion criteria

Children who had physical disability that could not permit collection of anthropometric data.

### Data collection

#### Assessment of household contextual characteristics

A structured questionnaire was used to obtain information on demographic characteristics, food handling practices (preparation, feeding, storage of raw ingredients, and surplus of cooked food); child feeding and dietary practices; diarrhoea incidence, and hygiene practices for children. Mothers or caregivers were the target respondents in this component of the study owing to the primary role they play in caring for children. Questions used were adopted with modification from literature [28–31]. The questionnaire was pre-tested in one sub-county for each of the districts where the study was conducted. However, these sub-counties were not included in the actual study. Questions that did not provide valid and reliable information were modified accordingly. The pre-test data were subjected to the Cronbach alpha test resulting into a reliability index of 0.70 and 0.77. This range of reliability has been considered acceptable for nutrition studies [32]. Data collection was undertaken by research assistants who were conversant with the local language (Ngakarimojong). Prior to data collection the assistants were trained on administration of the questionnaire.

#### Assessment of nutritional status of children under five

Weight for children under five was measured using a digital electronic mother child scale (ADE GmbH & Co., Hamburg, Germany) which allows for automated taring of the mother’s weight during the weighing process whereas height/length was measured using a Stadiometer. All measurements were carried out following guidelines for weight and height measurements from the World Health Organisation [33]. Age of the child was obtained from the birth/immunisation certificate or the child’s mother who was the study respondent.

### Data analysis

Data was coded, entered into SPSS version 20, exported and analysed using STATA Statistical package (Version 14). Weight, age, and height/length was entered into WHO Anthro software (Version 3.2.2) to obtain weight for age (WAZ), height for age (HAZ), and weight for height (WHZ) z scores. Distribution of categorical factors investigated were analysed using descriptive statistics (frequencies and percentages). Chi-square was used to test for the differences in categorical household characteristics and the prevalence of undernutrition indicators among under five age-groups across the three ecological zones. The level of statistical significance was set at 0.05. However, for predictors measured as continuous variables (household characteristics), data were checked for normality using Kolmogorov-Smirnov and Shapiro-Wilk tests. Owing to the non-normal distribution of the household characteristics (variables) that were continuous in nature, Kruskal-Wallis test was used to determine their differences among the agro-ecological zones followed by post hoc evaluation using Dunn’s test. The results were presented as median (Interquartile range). Factors associated with underweight, stunting, and wasting were determined using Binary Logistic Regression (BLR). Prior to the BLR, WAZ, HAZ, and WHZ were converted into dummy variables (1 = presence of underweight, stunting or wasting based on WAZ < -2SD, HAZ < -2SD, and WHZ < -2SD, respectively). As such, a child was considered underweight, stunted or wasted if the z score fell less than two standard deviations from the WHO median reference value for a population. Specifically, the study used the multi-variable binary logistic regression analysis and reported adjusted odds ratio (Results of the crude odds ratio have been presented in Appendix A-C of the supplementary material, Additional file 1). The pooled regression models were clustered by agroecology to account for agro-ecological differences. Prior to the BLR, the explanatory variables were screened for correlation using the pair-wise correlation test and only those that met the thresh-hold of having correlation coefficient of less than 0.7 [34] were included in the model (Table 1). Explanatory variables were also corrected for heteroskedasticity using the robust option in Stata to predict robust standard errors. All the models exhibited a non-significant goodness of fit test results, implying good model fit. Additionally, the wald test was significant for all the models indicating that the logit regression model was ideal for the analysis whereas the pseudo R^2^ for all the models ranged from 16 to 58% indicating that the predictor variables considered in this study account for 16–58% of the observed variation in underweight, stunting, and wasting across the agro-ecological zones. Predictor variables used in the regression analysis are presented in Table 1.

**Table 1:** Predictor variables used in the study

Predictors	Description of variables
Age of household head	Continuous variable in complete years
Number of children under five	Continuous variable
Sex of the child	Dummy, 1 = male, 0 = female
Weight	Continuous variable in kilograms
Age of child	Continuous variable in complete years
Height/ length	Continuous variable in centimeters
Education level of caregiver	Dummy, level of education, 1 = primary and above, 0 = no formal education
Occupation of household head	Dummy, main occupation of household head, 1 = crop farmer, 0 = others (pastoralists, agro-pastoralists, traders, casual labourers and civil servant)
Group membership of the parent	Dummy, 1 = yes, 0 = no
Training frequency on child feeding practices	Dummy, caregiver received training on feeding practices, 1 = at least once, 0 = never
Household expenses	Continuous variable in Uganda Shillings
Caregiver’s age	Continuous variable in complete years
Age at introduction of complementary food	Continuous variable in complete months
Drinking water treatment	Dummy, 1 = treated by boiling or tablets, 0 = not treated
Flour storage duration	Continuous variable in days
Consumption of leftover food	Dummy, 1 = more than 1 day, 0 = same day
Cooking duration	Continuous variable in complete hours
Sun drying of utensils	Dummy, utensils are dried under the sun after washing, 1 = yes, 0 = no
Breastfeeding	Dummy, child is still breastfeeding, 1 = yes, 0 = no
Washing of the child’s hand before feeding	Dummy, 1 = always, 0 = rarely and never
Caregiver hand washing after using latrine	Dummy, 1 = always, 0 = rarely and never
Diarrhoea in the last 14 days	Dummy, child had diarrhoea in the last 14 days, 1 = yes, 0 = no
Latrine ownership	Dummy, household owns a pit latrine, 1 = yes, 0 = no
Cereals storage (sacks)	Dummy, cereals stored in sacks, 1 = yes, 0 = no
Cereals storage (granary)	Dummy, cereals stored in granary, 1 = yes, 0 = no
Other methods (gunny bags, pots, on the floor)	Dummy, cereals stored using other methods, 1 = yes, 0 = no

## Results

### Household contextual characteristics of the study participants

A total of 240 households that were targeted responded to this study (Response rate = 100%). Contextual characteristics of the study participants are presented in Table 2.

**Table 2:** Distribution of household contextual characteristics of the study participants segregated by ecological zone

Household characteristics	PZ (***n*** = 55)		APZ (***n*** = 96)		AZ (***n*** = 89)		
	MD (IQR)	Mean ± SD	MD (IQR)	Mean ± SD	MD (IQR)	Mean ± SD	***p***-value
Age of household head (years)	35.0(16.0)	36.6 ± 11.4^b^	34.5(14.0)	37.2 ± 11.6^b^	45(20.0)	47.2 ± 14.1^a^	< 0.001*
Number of children under five	2(1.0)	1.6 ± 0.6^c^	2(1.0)	1.9 ± 1.1^b^	2(0.0)	2.1 ± 0.5^a^	0.0003*
Weight of the child (kg)	9.3(3.5)	9.3 ± 2.2^ab^	8.65(4.18)	10.0 ± 9.5^b^	10(4.0)	10.1 ± 2.8^a^	0.029*
Age of the child (months)	21(18.8)	23.8 ± 12.6^b^	18(13.50)	21.4 ± 11.9^b^	36(12.0)	30.9 ± 12.1^a^	< 0.001*
Height/ length of the child (cm)	79.75(15.3)	79.1 ± 13.7^ab^	75.75(14.4)	78.0 ± 11.3^b^	85.3(17.6)	79.2 ± 22.0^a^	0.005*
Cooking duration (hours)	3(2.0)	3.4 ± 1.9^b^	4(1.0)	4.3 ± 1.0^a^	4(1.0)	3.4 ± 0.8^b^	< 0.001*
Flour storage duration (days)	3(1.0)	3.2 ± 1.1^b^	6.5(4.0)	7.5 ± 6.2^a^	3(2.0)	3.5 ± 1.5^b^	< 0.001*
Household expenses (USD)	12.29(10.0)	19.6 ± 29.0^a^	13.66(10.7)	14.9 ± 9.6^a^	5.46(8.1)	7.2 ± 6.7^b^	< 0.001*
Caregiver’s age (years)	27(26.0)	33.6 ± 21.3^a^	8(3.0)	7.4 ± 6.9^c^	12(33.5)	25.7 ± 24.3^b^	< 0.001*
Age at introduction of complementary foods (months)	6(0.0)	6.2 ± 1.0^a^	6(0.0)	5.9 ± 1.1^ab^	6(0.0)	5.8 ± 0.8^b^	0.045*
**Categories**		**(%)**		**(%)**		**(%)**	
Sex of the child	Male	49.1		53.1		52.8	0.879
	Female	50.9		46.9		47.2	
Education level of caregiver	Primary and above	30.9		7.3		5.6	< 0.001*
	No formal education	69.1		92.7		94.4	
Occupation of household head	Crop farmer	67.3		82.3		49.4	< 0.001*
	Others^a^	32.7		17.7		50.6	
Group membership of the parent	(Yes)	32.7		33.3		24.7	0.390
Training on child feeding practices	At least once	34.5		88.5		60.7	< 0.001*
	Never	65.5		11.5		39.3	
Drinking water treatment	Treated by boiling or tablets	21.8		64.6		13.5	< 0.001*
	Not treated	78.2		35.4		86.5	
Consumption of leftover food	More than 1 day	34.5		67.7		10.1	< 0.001*
	Same day	65.5		32.3		89.9	
Sun drying of utensils	(Yes)	25.5		55.8		21.3	< 0.001*
Breastfeeding	(Yes)	61.8		72.9		44.9	0.001*
Washing of the child’s hand before feeding	Always	34.5		56.2		4.5	< 0.001*
	Rarely and never	65.5		43.8		95.5	
Caregiver hand washing after using latrine	Always	78.2		85.4		25.8	< 0.001*
	Rarely and never	21.8		14.6		74.2	
Diarrhoea in the last 14 days	(Yes)	27.3		54.2		57.3	0.001*
Latrine ownership	(Yes)	16.4		26.0		15.7	0.161
Cereals storage (Sacks)	(Yes)	56.4		38.5		75.3	< 0.001*
Cereals storage (Granary)	(Yes)	34.5		54.2		23.6	< 0.001*

^a^Others contain pastoralists, agro-pastoralists, traders, casual labourers and civil servant, 1USD = 3661.1 Uganda shillings as on 14th August 2020. PZ, APZ, and AZ denote pastoral, agro-pastoral, and agricultural ecological zones, respectively. SD, MD and IQR refer to the standard deviation, median and interquartile range, respectively. Mean values with different superscripts in each row are statistically significant (*p* < 0.05). * Shows statistical significance among ecological zones (*p* < 0.05)

All the household characteristics considered in this study significantly varied across the three agro-ecological zones except sex of the child, group membership of a child’s parent, and latrine ownership (*p* < 0.05) (Table 2). The median duration over which flour was stored in agro-pastoral communities were significantly higher than that of pastoral and agricultural communities by a factor of two (*p* < 0.05). Household expenses and age of the child in pastoral and agro-pastoral communities were significantly different from that of agricultural communities, however, they were not significantly different from each other (*p* < 0.001 and < 0.001, respectively). Both weight and height of the child were significantly higher in agricultural than agro-pastoral zone, however, that for pastoral zone was neither different from that of agricultural nor agro-pastoral zone (*p* = 0.029 and 0.005, respectively). The number of children under five was significantly different among all the agro-ecological zones (*p* < 0.001). Attainment of at least primary education among the caregivers varied from pastoral, agro-pastoral, and agricultural ecological zones in decreasing order of magnitude (*p* < 0.001). Attendance of training on child feeding practices and occurrence of diarrhoea followed similar patterns with increase from pastoral to agro-pastoral zone, however attendance of training on child feeding practices reduced in agricultural zone by 28% (*p* < 0.001) but diarrhoea increased by 3% instead (*p* = 0.001). However, drinking water treatment, washing of the child’s hand before feeding and washing of the hand by caregiver after using latrine followed a similar pattern but different from those of diarrhoea and training on child feeding practices. The extent of drinking water treatment among agro-pastoral communities was 43 and 51% higher than those in pastoral and agricultural communities (*p* < 0.001). Washing of the child’s hand before feeding (*p* < 0.001) and washing of the hand by caregiver after using latrine (*p* < 0.001) in agro-pastoral communities were 22, 7%, and 52, 60% higher than in pastoral and agricultural ecology, respectively.

### Nutritional status of children under five

Table 3 presents variation in prevalence of undernutrition indicators among children under five across ecological zones.

**Table 3:** Prevalence and distribution of nutritional status indicators among children under five segregated by ecology

Ecological zone	Prevalence of under nutrition (%)		
	Underweight (95% C.I)	Stunting (95% C.I)	Wasting (95% C.I)
	**6–11 months**		
Pastoral	50.0 (0.118–0.882)	50.0 (0.118–0.882)	33.3 (0.043–0.777)
Agro-pastoral	76.2 (0.528–0.918)	23.8 (0.082–0.472)	95.2 (0.762–0.999)
Agricultural	40.0 (0.053–0.853)	40.0 (0.053–0.853)	80 (0.284–0.995)
Chi-square (*p*-value)	2.743 (0.254)	2.743 (0.254)	13.967 (0.001^a^)
	**12–23 months**		
Pastoral	55.0 (0.315–0.769)	60.0 (0.361–0.809)	40.0 (0.191–0.639)
Agro-pastoral	51.5 (0.335–0.692)	48.5 (0.308–0.665)	60.6 (0.421–0.771)
Agricultural	78.6 (0.492–0.953)	50.0 (0.230–0.770)	78.6 (0.492–0.953)
Chi-square (*p*-value)	3.079 (0.215)	0.697 (0.706)	5.190 (0.075)
	**24–36 months**		
Pastoral	65.0 (0.408–0.846)	65.0 (0.408–0.846)	45.0 (0.231–0.685)
Agro-pastoral	22.6 (0.096–0.411)	51.6 (0.331–0.698)	19.4 (0.075–0.375)
Agricultural	38.9 (0.259–0.531)	48.1 (0.343–0.622)	38.9 (0.259–0.531)
Chi-square (*p*-value)	8.164 (0.017^a^)	1.147 (0.563)	4.275 (0.118)
	**37–59 months**		
Pastoral	42.9 (0.099–0.816)	42.9 (0.099–0.816)	14.3 (0.004–0.579)
Agro-pastoral	63.6 (0.308–0.891)	54.5 (0.234–0.833)	36.4 (0.109–0.692)
Agricultural	42.9 (0.198–0.701)	50.0 (0.247–0.753)	18.8 (0.040–0.456)
Chi-square (*p*-value)	1.211 (0.546)	0.234 (0.890)	1.543 (0.462)
	**Overall (6–59 months)**		
Pastoral (*n* = 55)	56.4 (0.423–0.697)	58.2 (0.441–0.713)	36.4 (0.238–0.504)
Agro-pastoral (*n* = 96)	49.0 (0.386–0.594)	44.8 (0.346–0.553)	52.1 (0.416–0.624)
Agricultural (*n* = 89)	46.1 (0.354–0.570)	48.3 (0.376–0.592)	43.8 (0.333–0.547)
Chi-square (*p*-value)	1.467 (0.480)	2.550 (0.279)	3.631 (0.163)

^a^ Shows statistical significance at α = 0.05 for association between agro-ecological zone and each undernutrition indicator. The age groups 6–11 months, 12–23 months, 24–36 months, and 37–59 months denote the under five age sub-groups. C.I denotes confidence interval

Generally, the prevalence of underweight, stunting, and wasting ranged from 36 to 58% across all ecological zones with no inter-ecological variation in magnitude (*p* > 0.05) (Table 3). However, their prevalence across the different under five age-groups among the three ecological zones varied significantly for underweight in children 24–36 months and wasting in children 6–11 months only (*p* < 0.05) but in none of the specific under five age groups for stunting. The prevalence of underweight and stunting were highest in pastoral ecology and least in agricultural and agro-pastoral ecological zones, respectively. However, prevalence of wasting was highest in agro-pastoral followed by agricultural and pastoral ecology in decreasing order of magnitude. Underweight peaked in children 24–36, 6–11, and 12–23 months in the pastoral, agro-pastoral, and agricultural ecology, respectively. Age group specific variation in the trend of stunting followed that of wasting for the pastoral ecology but not for the agro-pastoral and agricultural ecological zones. Stunting in agro-pastoral zone increased with age group and peaked at 37–59 months while in the agricultural zone, it peaked among the two age groups of 12–23 and 37–59 months. Wasting in both agricultural and agro-pastoral zones reduced with increasing age in a similar manner and peaked among children 6–11 months. A peculiar exception was the gradual rise in stunting among children 37–59 months in agro-pastoral zone. To the contrary, wasting among children in pastoral zone followed similar trends observed for underweight and stunting. The only exception was that the health indicator increased gradually and peaked among children 24–36 months.

### Predictors of nutritional status across ecological zones

#### Underweight

Factors associated with underweight across ecological zones are presented in Table 4.

**Table 4:** Predictors of underweight across ecological zones

Predictors	Pooled (*n* = 240)		Pastoral (***n*** = 55)		Agro-pastoral (***n*** = 96)		Agricultural (***n*** = 89)	
	Robust		Robust		Robust		Robust	
	Odds Ratio [95% C.I]	P > z	Odds Ratio [95% C.I]	P > z	Odds Ratio [95% C.I]	P > z	Odds Ratio [95% C.I]	P > z
Age of household head	0.998 [0.971–1.024]	0.885	0.977 [0.886–1.077]	0.640	0.991[0.943–1.042]	0.735	1.027 [0.985–1.071]	0.217
Sex of the child^a^	2.021 [0.981–4.165]	0.057	23.210 [3.144–171.368]	0.002*	4.394 [1.310–14.735]	0.017*	1.366 [0.452–4.126]	0.580
Height/ length	0.974 [0.934–1.016]	0.218	0.902 [0.789–1.030]	0.129	0.873 [0.807–0.943]	0.001*	0.991 [0.970–1.012]	0.404
Education level of caregiver^b^	2.818 [1.756–4.523]	< 0.001*	25.898 [2.080–322.477]	0.011*	1.396 [0.139–13.993]	0.777	2.456 [0.295–20.454]	0.406
Group membership^c^	0.539 [0.384–0.756]	< 0.001*	1.463[0.173–12.360]	0.727	0.446 [0.119–1.665]	0.229	0.659 [0.166–2.612]	0.553
Household expenses	0.646 [0.413–1.010]	0.055	0.130 [0.026–0.652]	0.013*	1.159 [0.434–3.097]	0.769	0.431 [0.226–0.825]	0.011*
Caregiver’s age	1.015 [1.002–1.028]	0.015*	0.993 [0.944–1.045]	0.791	1.117 [0.969–1.287]	0.128	1.022 [0.999–1.046]	0.062
Age at introduction of complementary food	1.383 [1.016–1.882]	0.039*	1.432 [0.679–3.022]	0.346	1.180 [0.645–2.160]	0.591	1.784 [0.871–3.654]	0.113
Drinking water treatment^d^	2.011 [0.876–4.618]	0.099	0.279 [0.009–8.857]	0.469	1.499 [0.461–4.868]	0.501	7.034 [1.302–37.986]	0.023*
Sun drying of utensils^e^	1.270 [0.851–1.897]	0.242	3.183 [0.351–28.904]	0.304	0.964 [0.290–3.205]	0.953	1.843 [0.403–8.437]	0.431
Consumption of leftover food^f^	1.334 [0.825–2.158]	0.240	0.879 [0.021–36.657]	0.946	3.524 [0.834–14.893]	0.087	1.119 [0.151–8.310]	0.912
Flour storage duration	0.926 [0.875–0.980]	0.008*	1.575 [0.478–5.187]	0.455	0.805 [0.692–0.935]	0.005*	1.080 [0.714–1.634]	0.714
Cooking duration	1.345 [1.238–1.461]	< 0.001*	2.206 [1.168–4.167]	0.015*	1.246 [0.695–2.234]	0.460	2.192 [0.940–5.109]	0.069
Breastfeeding^g^	1.940 [0.560–6.719]	0.296	2.388 [0.416–13.700]	0.32	0.211 [0.048–0.923]	0.039*	3.461 [1.148–10.431]	0.027*
Latrine ownership^h^	1.510 [0.545–4.182]	0.428	13.011 [0.626–270.533]	0.097	0.346 [0.069–1.743]	0.198	5.679 [1.293–24.940]	0.021*
Cereals storage (sacks)^i^	0.521 [0.049–5.584]	0.590	1.854 [0.108–31.913]	0.671	0.017 [0.002–0.160]	< 0.001*		
Cereals storage (granary)^j^	0.749 [0.140–4.016]	0.736	0.261[0.013–5.123]	0.377	0.064 [0.007–0.578]	0.014*		
Constant	17.275 [0.082–3647.317]	0.297	3.42E+ 09 [1.325- 8.84E+ 18]	0.047	83,342.88 [1.74615- 3.98E+ 09]	0.039	0.431[0.000–1397.407]	0.838
Wald chi^2^(19)	48.51		39.22		30.66		25.23	
Prob > chi^2^	0.00		0.002		0.022		0.047	
Log likelihood	−139.525		−19.156		- 43.200		- 47.002	
Pseudo R^2^	0.158		0.484		0.351		0.235	

a, 1 = male, 0 = female; b, 1 = primary and above, 0 = no formal education; c, 1 = yes, the parent belongs to a group, 0 = no; d, 1 = treated by boiling or tablets, 0 = not treated; e, 1 = yes, 0 = no; f, 1 = more than 1 day, 0 = same day; g, 1 = yes, the child is still breastfeeding, 0 = No; h; 1 = yes, 0 = no; i; 1 = yes, 0 = no; and j, 1 = yes, 0 = no. Other storage methods were considered as base category for storage methods. C.I denotes confidence interval. * Shows statistical significance (*p* < 0.05)

Generally, underweight was significantly dependent on sex and height/length of the child, education level of caregiver, household expenses, drinking water treatment, flour storage duration, cooking duration, breastfeeding, latrine ownership, cereal storage methods, group membership, caregiver’s age and age at introduction of complementary foods with disparities across zones (Table 4). Being a male increased the odds of being underweight by 23 and 4 times in pastoral (OR 23.210; 95% C. I 3.144–171.368) and agro-pastoral (OR 4.394; 95% C.I 1.310–14.735) but not in agricultural ecological zone (Table 4). Household expenses reduced the likelihood of a child being underweight by 0.1 and 0.4 times in pastoral (OR 0.130; 95% C.I 0.026–0.652) and agricultural zones (OR 0.431; 95% C.I 0.226–0.825) but not in agro-pastoral ecology. A breastfed child was 0.2 times less likely to be underweight in agro-pastoral zone (OR 0.211; 95% 0.048–0.923) whereas to the contrary a breastfed child in agricultural zone was 3.5 times more likely to be underweight (OR 3.461; 95% C.I 1.148–10.431). Cooking duration and education level of the respondent being at least primary increased the likelihood of being underweight by 2 (OR 2.206; 95% C.I 1.168–4.167) and 25-fold (OR 25.898; 95% C.I 2.080–322.477) in pastoral zone only (Table 4). Child height/length (OR 0.873; 95% C.I 0.807–0.943), flour storage duration (OR 0.805; 95% C.I 0.692–0.935) and cereal storage in sacks (OR 0.017; 95% C.I 0.002–0.160) and granary (OR 0.064; 95% C.I 0.007–0.578) reduced the likelihood of underweight in agro-pastoral zone only by magnitude ranging from 0.01 to 0.8-fold. Latrine ownership (OR 5.679; 95% C.I 1.293–24.940) and drinking water treatment (OR 7.034; 95% C.I 1.302–37.986) increased underweight likelihood by 5.7 and 7 times in agricultural zone only, respectively. Lastly, group membership of the parent significantly reduced the likelihood of being underweight by half (OR 0.539; 95% C.I 0.384–0.756) whereas caregiver’s age (OR 1.015; 95% C.I 1.002–1.028) and age at introduction of complementary foods resulted into increase in the likelihood of being underweight (OR 1.383; 95% C.I 1.016–1.882) irrespective of agro-ecological zone.

#### Stunting

Predictors of stunting among children under five across ecological zones are presented in Table 5.

**Table 5:** Predictors of stunting across ecological zones

Predictors	Pooled (***n*** = 240)		Pastoral (***n*** = 55)		Agro-pastoral (***n*** = 96)		Agricultural (***n*** = 89)	
	Robust		Robust		Robust		Robust	
	Odds Ratio [95% C.I]	P > z	Odds Ratio [95% C.I]	P > z	Odds Ratio [95% C.I]	P > z	Odds Ratio [95% C.I]	P > z
Number of children under five	1.059 [0.711–1.578]	0.779	0.017 [0.000–0.61]	0.026*	1.789 [0.836–3.828]	0.134	1.202 [0.491–2.939]	0.687
Weight	0.660 [0.580–0.752]	< 0.001*	0.468 [0.218–1.00]	0.052	0.604 [0.443–0.822]	0.001*	0.501 [0.382–0.658]	< 0.001*
Sex of the child^a^	2.108 [0.829–5.361]	0.117	9.098 [1.888–33.558]	0.011*	7.242 [2.009–26.108]	0.002*	0.786 [0.265–2.330]	0.664
Occupation of household head^b^	0.459 [0.159–1.319]	0.148	0.004 [0.000–0.10]	0.001*	2.981 [0.637–13.945]	0.165	0.469 [0.121–1.822]	0.274
Training frequency on child feeding practices^c^	0.459 [0.285–0.740]	0.001*	0.256 [0.038–1.73]	0.162	0.359 [0.055–2.343]	0.284	0.678 [0.208–2.203]	0.518
Education level of caregiver^d^	0.885 [0.706–1.108]	0.287	0.070 [0.004–1.12]	0.06	0.359 [0.054–2.410]	0.292	0.426 [0.067–2.725]	0.368
Group membership^e^	0.535 [0.156–1.843]	0.322	5.042 [0.285–8.903]	0.007*	0.122 [0.032–0.459]	0.002*	0.398 [0.091–1.737]	0.22
Caregiver’s age	1.024 [1.010–1.038]	0.001*	1.015 [0.960–1.07]	0.600	1.028 [0.961–1.099]	0.422	1.019 [0.997–1.042]	0.09
Flour storage duration	1.112 [1.057–1.170]	< 0.001*	5.582 [1.158–26.92]	0.032*	1.187 [1.050–1.342]	0.006*	0.945 [0.610–1.465]	0.801
Cooking duration	1.158 [1.029–1.305]	0.015*	2.143 [0.860–5.34]	0.102	0.970 [0.504–1.865]	0.927	2.787 [1.254–6.195]	0.012*
Breastfeeding^f^	0.341 [0.127–0.916]	0.033*	1.660 [0.123–22.33]	0.702	0.088 [0.017–0.459]	0.004*	0.255 [0.060–1.086]	0.065
Consumption of leftover food^g^	0.953 [0.910–1.000]	0.049*	0.823 [0.564–1.20]	0.311	0.772 [0.606–0.983]	0.036*	0.764 [0.583–1.002]	0.051
Caregiver hand washing after using latrine^h^	1.698 [1.086–2.654]	0.020*	12.498 [1.112–45.774]	0.005*	1.821 [0.421–7.875]	0.422	19.436 [0.526–718.094]	0.107
Diarrhoea in the last 14 days^i^	0.902 [0.771–1.054]	0.194	2.324 [1.184–5.572]	< 0.001*	0.957 [0.676–1.356]	0.805	0.941 [0.686–1.291]	0.706
Cereals storage (sacks)^j^	0.256 [0.059–1.108]	0.068	0.068 [0.002–1.94]	0.116	0.079 [0.011–0.563]	0.011*		
Cereals storage (granary)^k^	0.225 [0.031–1.631]	0.140	0.854 [0.040–18.47]	0.92	0.054 [0.005–0.602]	0.018*		
Constant	213.184 [101.725–446.766]	< 0.001	180,264.1 [0.604973–5.37E+ 10]	0.06	695.042 [2.502–193,040.5]	0.023	208.9424 [0.93923–46,481.65]	0.053
Wald chi^2^(19)	58.3		27.45		32.83		33.26	
Prob > chi^2^	0		0.037		0.008		0.003	
Log likelihood	−130.414		−15.600		−40.078		−41.349	
Pseudo R^2^	0.216		0.583		0.393		0.329	

a, 1 = male, 0 = female; b, 1 = crop farmer, 0 = others (pastoralists, agro-pastoralists, traders, casual labourers and civil servant); c, 1 = at least once, 0 = never; d, 1 = primary and above, 0 = no formal education; e, 1 = yes, the parent belongs to a group, 0 = no; f, 1 = yes, the child is still breastfeeding, 0 = No; g, 1 = more than 1 day, 0 = same day; h, 1 = always, 0 = rarely and never; i, 1 = yes, 0 = no; j, 1 = yes, 0 = no; and k, 1 = yes, 0 = no. Other storage methods were considered as base category for storage methods. C.I denotes confidence interval. * Shows statistical significance (*p* < 0.05)

The undernutrition indicator was predicted by several factors and exhibited disparity across ecological zones as in the case of underweight (Table 5). The likelihood of stunting reduced by 0.6 (OR 0.604; 95% C.I 0.443–0.822) and 0.5 times (OR 0.501; 95% C.I 0.382–0.658) with weight of the child in agro-pastoral and agricultural zone, respectively. Male children were 9 and 7 times more likely to be stunted than their female counter parts in both pastoral (OR 9.098; 95% C.I 1.888–33.558) and agro-pastoral (OR 7.242; 95% C.I 2.009–26.108) ecology. Similarly, flour storage duration was associated with 5.6 and 1.2 times increase in the likelihood of stunting in pastoral (OR 5.582; 95% C.I 1.158–26.92) and agro-pastoral (OR 1.187; 95% C.I 1.050–1.342) ecology, respectively. Children under five belonging to parents who were members of a group within the community were 5 times more likely to be stunted in pastoral zone (OR 5.042; 95% C.I 0.285–8.903) whereas to the contrary children under five whose mothers belonged to groups were 0.1 times less likely to be stunted (OR 0.122; 95% C.I 0.032–0.459) in agro-pastoral zone. Breastfed children and those who consumed leftover food in agro-pastoral zone only were 0.1 (OR 0.088; 95% C.I 0.017–0.459) and 0.8 (OR 0.772; 95% C.I 0.606–0.983) times less likely to be stunted. The number of factors predicting stunting in a single agro-ecological zone was highest in agro-pastoral, followed by pastoral, and least in agricultural zone in decreasing order of magnitude (Table 5). Storage of cereals in both sacks and granaries reduced the likelihood of stunting by 0.1 times in agro-pastoral zone (OR 0.079; 95% C.I 0.011–0.563 and OR 0.054; 95% C.I 0.005–0.602, respectively). Increase in cooking duration by 1 hour increased the likelihood of stunting by 2.8 times (OR 2.787; 95% C.I 1.254–6.195) in agricultural zone only. The number of children under five in a household (OR 0.017; 95% C.I 0.000–0.61), being a crop farmer as occupation of the household head (OR 0.004; 95% C.I 0.000–0.10) reduced the likelihood of stunting in pastoral zone only. Lastly, a child who had diarrhoea in the last 2 weeks was 2.3 times more likely to be stunted (OR 2.324; 95% C.I 1.184–5.572) whereas hand washing by the caregiver after using the latrine always in pastoral communities increased the likelihood of stunting among children under five instead (OR 12.498; 95% C.I 1.112–45.774). Lastly, attendance of training on child feeding practices at least once by the parent reduced the likelihood of stunting by half (OR 0.459; 95% C.I 0.285–0.740) whereas increase in caregiver’s age slightly increased the likelihood of stunting (OR 1.024; 95% C.I 1.010–1.038) in general irrespective of agro-ecological zone.

#### Wasting

The factors that predicted wasting among children under five are presented in Table 6.

**Table 6:** Predictors of wasting among children under five across ecological zones

Predictors	Pooled (***n*** = 240)		Pastoral (***n*** = 55)		Agro-pastoral (***n*** = 96)		Agricultural (***n*** = 89)	
	Robust		Robust		Robust		Robust	
	Odds Ratio [95% C.I]	P > z	Odds Ratio [95% C.I]	P > z	Odds Ratio [95% C.I]	P > z	Odds Ratio [95% C.I]	P > z
Age of household head	0.994 [0.960–1.029]	0.731	0.962 [0.896–1.034]	0.293	0.964 [0.917–1.014]	0.160	1.015 [0.975–1.058]	0.470
Number of children under five	0.894 [0.821–0.974]	0.010*	0.477 [0.149–1.528]	0.213	0.843 [0.502–1.415]	0.518	0.648 [0.247–1.700]	0.378
Age of child	0.952 [0.934–0.971]	< 0.001*	0.982 [0.919–1.049]	0.587	0.914 [0.853–0.979]	0.011*	0.902 [0.841–0.968]	0.004*
Sex of the child^a^	1.727 [0.829–3.598]	0.145	3.993 [0.932–17.104]	0.062	4.961 [1.545–15.933]	0.007*	0.818 [0.279–2.398]	0.714
Education level of caregiver^b^	1.449 [0.304–6.896]	0.641	7.205 [0.957–54.234]	0.055	0.194 [0.032–1.178]	0.075	2.640 [0.331–21.048]	0.359
Household expenses	0.474 [0.364–0.619]	< 0.001*	0.177 [0.052–0.600]	0.005*	0.548 [0.204–1.473]	0.233	0.507 [0.284–0.904]	0.021*
Age at introduction of complementary food	1.054 [0.874–1.271]	0.581	2.012 [1.114–3.633]	0.020*	0.982 [0.643–1.500]	0.934	1.006 [0.473–2.138]	0.988
Drinking water treatment^c^	3.984 [2.570–6.176]	< 0.001*	6.101 [1.113–33.445]	0.037*	9.207 [2.395–35.394]	0.001*	16.344 [3.052–87.534]	0.001*
Consumption of leftover food^d^	0.726 [0.395–1.334]	0.302	2.171 [0.389–12.108]	0.377	0.234 [0.073–0.750]	0.015*	0.545 [0.084–3.510]	0.523
Breastfeeding^e^	1.213 [0.759–1.940]	0.420	1.470 [0.317–6.805]	0.623	0.774 [0.176–3.405]	0.735	1.201 [0.296–4.877]	0.798
Washing of the child’s hand before feeding^f^	0.933 [0.297–2.920]	0.905	15.898 [2.154–117.311]	0.007*	0.171 [0.026–1.148]	0.069	1.535 [0.384–6.130]	0.544
Constant	3546.552 [1578.626-7967.706]	< 0.001	110,171.9 [1.308-9.28E+ 09]	0.045	30,202.38 [0.243-3.75E+ 09]	0.085	15,875.67 [2.839–8.88E+ 07]	0.028
Wald chi^2^(19)	47.8		18.08		27.75		26.98	
Prob > chi^2^	0		0.080		0.004		0.005	
Log likelihood	− 137.099		−24.775		−45.136		−45.703	
Pseudo R^2^	0.171		0.313		0.321		0.251	

a, 1 = male, 0 = female; b, 1 = primary and above, 0 = no formal education; c, 1 = treated by boiling or tablets, 0 = not treated; d, 1 = more than 1 day, 0 = same day; e, 1 = yes, 0 = no; and f, 1 = always, 0 = rarely and never; *C.I* denotes confidence interval; * shows statistical significance (*p* < 0.05)

The relationship between wasting and the factors studied followed a similar trend as in the case of stunting. However, treatment of drinking water by boiling or using tablets increased the odds of wasting by 6.1, 9.2 and 16.3 times in pastoral, agro-pastoral and agricultural zone respectively (OR 6.101; 95% C.I 1.113–33.445, OR 9.207; 95% C.I 2.395–35.394, OR 16.344; 95% C.I 3.052–7.534 for pastoral, agro-pastoral, and agricultural zone, respectively). A child whose hand was washed before feeding in pastoral zone only was 15.9 times more likely to be wasted (OR 15.898; 95% C.I 2.154–117.311). Children from households that incurred more expenses in pastoral and agricultural but not in agro-pastoral zone were 0.2 (OR 0.177; 95% C.I 0.052–0.600) and 0.5 times (OR 0.507; 95% C.I 0.284–0.904) less likely to be wasted, respectively. Wasting likelihood reduced by 0.9 times with increase in age in both agro-pastoral (OR 0.914; 95% C.I 0.853–0.979) and agricultural (OR 0.902; 95% C.I 0.841–0.968) but not in pastoral zone. Age at introduction of complementary food in pastoral zone increased the likelihood of wasting by 2 times (OR 2.012; 95% C.I 1.114–3.633). Being a male child increased the wasting likelihood by 5 times (OR 4.961; 95% C.I 1.545–15.933) whereas consumption of leftover food after more than a day in agro-pastoral zone reduced the likelihood of wasting by 0.2 times (OR 0.234; 95% C.I 0.073–0.750). Irrespective of agro-ecological zone, number of children under five reduced the likelihood of wasting by 0.9 times (OR 0.894; 95% C.I 0.821–0.974). However, there was no factor associated with wasting in agricultural zone only.

## Discussion

Investigating the prevalence of underweight, stunting, and wasting in children under five among vulnerable communities typical of Karamoja sub-region is essential for designing appropriate public health interventions [35]. Considering that previous studies in Karamoja sub-region characterised undernutrition in general [36, 37] despite agro-ecological disparity, this study investigated undernutrition as affected by agroecology that determines the livelihood activities. Considering the fact that nutrition in Karamoja sub-region, one of the most food insecure parts of Eastern Africa depend on agriculture, the differences in agroecology provides opportunity for policy intervention to reduce undernutrition in respective agroecology.

In this study, all indicators of undernutrition were above WHO acceptable level for the respective indicators in a given community (< 10, < 20, and < 5% for underweight, stunting, and wasting, respectively) [13] and at least 1.4 times higher than levels reported in the same area 3 years earlier which stood at 26, 35, and 10%, for underweight, stunting, and wasting, respectively [10]. This indicates that undernutrition among children under five is endemic in Karamoja sub-region and is on the rise making it of public health concern. These results bring into question, the effectiveness of various intervention efforts by government and non-governmental organisations at mitigating child undernutrition situation in the sub-region.

Disparity in the prevalence of underweight, stunting, and wasting across the three agro-ecological zones at specific under five age groups (Table 3) underscores the significance of agroecology in the occurrence of undernutrition among children in food insecure localities such as Karamoja sub-region. Identification of age groups at highest risk of underweight, stunting, and wasting is essential for tailoring interventions aimed at reducing undernutrition in children under five [38]. In the current study, underweight peaked at 24–36, 6–11, 12–23 months in pastoral, agro-pastoral, and agricultural ecology, respectively. However, Habaasa [39] reported higher risk of underweight among children 6–11 months in Nakaseke and Nakasongola communities in mid-central Uganda while in another study by Khan et al. [40], underweight in children peaked at 24–35 months in Singh region of Pakistan. It is important to note that geographically this study and that of Habaasa [39] were conducted in the cattle corridor of Uganda. The disparity between the findings of this study and that of Habaasa [39] could be attributed to the predisposing risk factors for underweight such as exclusive breastfeeding, income, and type of weaning foods that are dependent on predominant livelihood activities along the cattle corridor [16].

The peaking of stunting at 12–23 months in agricultural zone (Table 3) concurs with the findings of Dake et al. [38]. On the other hand, wasting peaked at 6–11 months which concurs with the findings of Garenne et al. [41] for agro-pastoral and agricultural zones only. These observations signifies the disparity in the occurrence of age specific risk factors of stunting (for example income, pre-lacteal feeding, age) and wasting (for example diarrhoea, poor breastfeeding, limited dietary diversity) among children under five across agro-ecological zones of Karamoja sub-region as dictated by the prevailing ecological livelihoods [12, 42]. By implication, the results of this study bring to the fore, that interventions designed to address undernutrition among children in localities such as the Karamoja sub-region should take into consideration agroecology and the different age groups at which each of the undernutrition indicator peaks.

Undernutrition is the major risk factor for morbidity and mortality among children under five in developing countries [42, 43]. This makes understanding the factors that lead to undernutrition very vital in agro-ecologically based localities such as the Karamoja sub-region. Indeed, results show that disparity in factors that predict underweight, stunting, and wasting in children under five exists among households in the pastoral, agro-pastoral and agricultural agro-ecological zones.

The odds for all the undernutrition indicators increased with sex of the child being male in pastoral and agro-pastoral zones except for wasting that occurred in pastoral zone only. Disparity in underweight, stunting, and wasting based on sex of the child could be due to the variation in nutritionally associated cultural practices accorded to children of different sexes such as complementary and weaning feeding practices [44, 45]. Previous studies have reported disparity in feeding and care practices accorded to children of different sexes in India [44] and Rwanda [46] although ecological zone was not considered.

Group membership of the parent reduced the likelihood of underweight irrespective of ecology and that of stunting in agro-pastoral but increased that of stunting in pastoral zone instead. The disparity reflecting group membership influence on undernutrition indicators across agroecology could be due to the extent of information sharing among group members on child feeding and care practices for appropriate nutrition behaviour [47] or extent to which resources obtained from the groups are channeled to child nutrition and care.

The flour storage duration increased the stunting likelihood in pastoral and agro-pastoral zone but reduced that for underweight in agro-pastoral zone instead. This observation could be due to the occurrence of mycotoxins and aflatoxins in particular [48] that compromises linear growth in children [49]. However, the reduced risk of underweight shows that in the short run the flour provides nutrients in the diet despite becoming a risk factor for stunting in the long run.

Cooking duration was associated with increased likelihood of being underweight in pastoral and stunting in agricultural zone. Longer cooking duration often leads to loss of heat labile nutrients and consequently low nutrient intake [50]. The differential influence of cooking duration on underweight and stunting across the two agro-ecological zones could be due to the predominance of diet by plant-based foods in the agricultural zone [50].

Breastfeeding is known to improve immunity and increase nutrient intake among children [51, 52]. The reduced odds of underweight and stunting in agro-pastoral zone and increased odds of underweight in agricultural zone among breastfed children suggest existence of appropriate breastfeeding practices in agro-pastoral but not in agricultural zone. Similarly, reduced odds of underweight and stunting with cereal storage in granary and sacks in agro-pastoral zone only could be attributed to the appropriate postharvest handling practices during storage among agro-pastoralist thereby reducing the occurrence of mycotoxins that is associated with underweight and stunting [48, 53].

Caregiver’s age was associated with slight increase in the likelihood of underweight and stunting irrespective of agro-ecological zones. This shows that age disparity of caregivers plays a role towards undernutrition in general but not with respect to specific ecology. Drinking water treatment increased the likelihood of wasting in all agroecology and for underweight in only agricultural zone. These scenarios could be explained by microbial contamination (e.g with diarrheagenic *E. coli*) of water post preparation due to unhygienic practices, a factor known to cause underweight [16] and wasting when prolonged [53]. Financial resources provide means to obtain food and other essential commodities for the wellbeing of a child [54]. As such, the positive influence of household expenses on underweight and wasting in only pastoral and agricultural communities could be explained by variation in the extent to which households in the respective agro-ecological zones spend their financial resources on accessing nutritious foods for children under five [55].

The odds of wasting increased with age at introduction of complementary food in pastoral zone only and that of underweight irrespective of agroecology. These could be attributed to late or early introduction of complementary food against the WHO recommendation of introducing complementary foods at 6 months observed in the sub-region (Table 2). Reduced odds of wasting and stunting with consumption of leftover food after more than 1 day in agro-pastoral zone only could be explained by increased nutrient intake among children [56, 57].

Increased odds of underweight due to latrine ownership in agricultural and education of caregiver in pastoral zones only could be explained by non-adherence to latrine use despite ownership and limited child care knowledge that might be associated with primary level of education [16]. The reduced odds of underweight with increase in height/length of the child from agro-pastoral zone only could be due to variation in transgenerational thinness of different pastoral groups [16, 43]. Training frequency on child feeding practices irrespective of agroecology, household head being a crop farmer and number of children under five in pastoral zone as well as weight of the child in agro-pastoral zone were associated with reduced stunting likelihood. These observations could be explained by the fact that weight is a component of linear growth [58] and the extent to which households allocate resources for the well-fare of children under five [21, 59].

Increased odds of stunting with caregiver hand washing after visiting latrine and diarrhoea in pastoral zone only suggest that there is high water and environmental contamination by pathogenic microorganisms in pastoral zone that is attributable to open defecation [23, 38]. Furthermore, increase in wasting likelihood in pastoral zone with the practice of washing of a child’s hand before feeding further demonstrates the extent of water contamination in pastoral zone [23]. Lastly, reduced likelihood of wasting with age of child in agricultural and agro-pastoral zones only could be attributed to disparity in nutrient density of foods and nutrient intake among children in different ecological zones [60, 61]. Therefore, ecology specific household contextual characteristics with respect to food handling, child feeding practices, and demographic factors should be considered in designing appropriate interventions to address undernutrition in children under five.

## Limitations

Whereas the findings of this study provide vital insights into the disproportionate agro-ecological based prevalence of undernutrition in children under 5 years and the associated factors, some limitations suffice. First, the small sample size of respondents used in each agroecology might limit the generalizability of the study as previously reported in other cross-sectional studies on nutrition [62]. Therefore, a large-scale future study is recommended to gain deeper understanding of the contextual differences among the agro-ecological zones in predicting undernutrition in children under five in Karamoja sub-region. Secondly, the selection of the districts with highest level of undernutrition in each agro-ecological zones could also limit the generalizability of the study findings in locations that are different from Karamoja region.

## Conclusions

Children under five across all ecological zones of the Karamoja sub-region of Uganda suffer from multiple forms of undernutrition at levels beyond acceptable threshold with disparity in prevalence of undernutrition indicators and age of peaking across different ecological zones. This form of malnutrition is associated with household contextual characteristics of unhygienic food handling, child feeding, and dietary practices and negative demographic factors across the respective ecological zones. Therefore, interventions targeting undernutrition among children under five in Karamoja sub-region and similar localities elsewhere should consider differences in agroecology, household contextual characteristics and age of occurrence of undernutrition.

## Supplementary Information

**Additional file 1.** Disparity in prevalence and predictors of undernutrition in children under five among agricultural, pastoral, and agro-pastoral ecological zones of Karamoja sub-region, Uganda: A cross sectional study. Appendices A-C provide results of the crude odds ratio for predictors of underweight, stunting and wasting across ecological zones.

## Data Availability

The datasets used and/or analysed during the current study are available from the corresponding author on reasonable request.

## Abbreviations

BBNC: Bangalore Boston Nutrition Collaborative
CAO: Chief Administrative Officer
CESAAM: Centre of Excellence in Sustainable Agriculture and Agribusiness Management
GAM: Global Acute Malnutrition
GUREC: Gulu University Research Ethics Committee
HAZ: Height for age z scores
NCHS: National Centre for Health Statistics
SSA: Sub-Saharan Africa
WAZ: Weight for age z scores
WHO: World Health Organisation
WHZ: weight for height z scores
