# Supplementary material for: Disparity in prevalence and predictors of undernutrition in children under five among agricultural, pastoral, and agro-pastoral ecological zones of Karamoja sub-region, Uganda: a cross sectional study
Source: BMC Pediatr. 2022 May 30;22:316. doi: 10.1186/s12887-022-03363-6 (PMC9150356; doi:10.1186/s12887-022-03363-6)
Supplement: Supplementary file 1 — Additional file 1. Disparity in prevalence and predictors of undernutrition in children under five among agricultural, pastoral, and agro-pastoral ecological zones of Karamoja sub-region, Uganda: A cross sectional study. Appendices A-C provide results of the crude odds ratio for predictors of underweight, stunting and wasting across ecological zones. [file 12887_2022_3363_MOESM1_ESM.pdf]

Appendix A. Crude odds ratio for predictors of underweight across ecological zones

Predictors	Pooled (n=240)		Pastoral (n=55)		Agro-pastoral (n=96)		Agricultural (n=89)	
	Crude Odds Ratio [95% C.I]	P>z	Crude Odds Ratio [95% C.I]	P>z	Crude Odds Ratio [95% C.I]	P>z	Crude Odds Ratio [95% C.I]	P>z
Age of household head	0.984 [0.962-1.007]	0.164	0.947 [0.898-0.999]	0.048*	0.983 [0.945-1.022]	0.390	1.000 [0.971-1.031]	0.954
Sex of the child[a]	1.497 [0.840-2.665]	0.170	8.307 [0.766-23.945]	0.137	1.977 [0.872-4.482]	0.103	0.888 [0.384-2.057]	0.783
Height/ length	0.972 [0.935-1.011]	0.162	0.945 [0.895-0.997]	0.040*	0.929 [0.883-0.978]	0.005*	0.989 [0.968-1.011]	0.334
Education level of caregiver[b]	1.782 [0.870-3.653]	0.115	7.4 [0.699-26.236]	0.164	0.767 [0.161-3.656]	0.739	1.816 [0.285-11.553]	0.528
Group membership[c]	0.687 [0.439-1.076]	0.101	0.682 [0.217-2.140]	0.512	0.497 [0.208-1.190]	0.117	0.968 [0.366-2.559]	0.947
Household expenses	0.816 [0.726-0.918]	0.001*	0.648 [0.364-1.153]	0.140	0.720 [0.407-1.273]	0.258	0.769 [0.492-1.201]	0.248
Caregiver's age	1.006 [1.005-1.007]	0.000*	1.000 [0.975-1.026]	0.982	1.037 [0.978-1.099]	0.128	1.007 [0.990-1.025]	0.410
Age at introduction of complementary food	1.416 [1.186-1.690]	0.000*	1.223 [0.709-2.107]	0.469	1.314 [0.892-1.935]	0.167	1.750 [0.832- 3.682]	0.140
Drinking water treatment[d]	1.477 [0.676-3.227]	0.328	2.864 [0.672-12.211]	0.155	0.938 [0.404-2.174]	0.880	4.219 [1.050-16.952]	0.043*
Sun drying of utensils[e]	0.826 [0.477-1.430]	0.494	0.708 [0.207-2.423]	0.582	0.607 [0.269-1.371]	0.230	1.398 [0.503-3.886]	0.521
Consumption of leftover food[f]	0.939 [0.440-2.006]	0.872	0.356 [0.110-1.149]	0.084	1.719 [0.720-4.100]	0.222	1.076 [0.267-4.336]	0.918
Flour storage duration	0.951 [0.901-1.004]	0.070	1.579 [0.929-2.684]	0.091	0.920 [0.864-0.980]	0.010*	1.115 [0.838-1.484]	0.455
Cooking duration	1.361 [1.172-1.581]	0.000*	1.739 [1.186-2.552]	0.005*	1.266 [0.816-1.964]	0.293	1.444 [0.887-2.352]	0.139
Breastfeeding[g]	2.109 [1.665- 2.671]	0.000*	2.444 [0.794-7.526]	0.119	1.794 [0.712-4.519]	0.215	2.330 [0.987-5.503]	0.054
Latrine ownership[h]	1.398 [0.575-3.398]	0.460	3.208 [0.592-17.388]	0.176	0.764 [0.304-1.919]	0.567	2.419 [0.734-7.965]	0.146
Cereals storage (sacks)[i]	0.715 [0.414-1.236]	0.230	1.583 [0.533-4.701]	0.408	0.4795 [0.206-1.116]	0.088	0.6359[0.240-1.684]	0.362
Cereals storage (granary)[j]	1.363 [0.917- 2.026]	0.126	0.7936 [0.257-2.453]	0.688	1.535 [0.681-3.459]	0.301	1.793 [0.663-4.847]	0.250
Constant	26.226 [0.293-2346.82]	0.154	3.42E+09 [1.325-8.84E+18]	0.047	83342.88 [1.74615-3.98E+09]	0.039	0.431[0.000-1397.407]	0.838
Wald chi^2(19)	48.51		39.22		30.66		25.23	
Prob > chi^2	0.00		0.002		0.022		0.047	

Log likelihood	-139.525	-19.156	- 43.200	- 47.002
Pseudo R^2	0.158	0.484	0.351	0.235

a, 1=male, 0=female; b, 1=primary and above, 0=no formal education; c, 1=yes, the parent belongs to a group, 0=no; d, 1=treated by boiling or tablets, 0= not treated; e, 1=yes, 0=no; f, 1=more than 1 day, 0=same day; g, 1= yes, the child is still breastfeeding, 0=No; h; 1=yes, 0=no; i; 1=yes, 0=no; and j, 1=yes, 0=no. Other storage methods were considered as base category for storage methods. C.I denotes confidence interval. * Shows statistical significance ($p<0.05$).

Appendix B. Crude odds ratio for predictors of stunting across ecological zones

Predictors	Pooled (n=240)		Pastoral (n=55)		Agro-pastoral (n=96)		Agricultural (n=89)	
	Crude Odds Ratio [95% C.I]	P>z	Crude Odds Ratio [95% C.I]	P>z	Crude Odds Ratio [95% C.I]	P>z	Crude Odds Ratio [95% C.I]	P>z
Number of children under five	0.925 [0.694-1.231]	0.591	0.603 [0.254-1.433]	0.252	1.048 [0.714-1.538]	0.811	1.010 [0.479-2.127]	0.980
Weight	0.800 [0.668-0.957]	0.015 *	0.702 [0.546-0.903]	0.006 *	0.894 [0.782-1.022]	0.100	0.703 [0.588-0.841]	0.000 *
Sex of the child[a]	1.551 [1.002-2.399]	0.049 *	2 [0.665-6.011]	0.217	2.039 [0.893-4.657]	0.091	1.054 [0.456-2.436]	0.902
Occupation of household head[b]	0.514 [0.259-1.021]	0.057	0.159 [0.031-0.812]	0.027 *	0.792 [0.212-2.956]	0.728	0.669 [0.277-1.615]	0.372
Training frequency on child feeding practices[c]	0.608 [0.419-0.881]	0.009 *	0.707 [0.228-2.195]	0.549	0.420 [0.113-1.554]	0.194	0.814 [0.346-1.917]	0.638
Education level of caregiver[d]	1.315 [0.919-1.882]	0.134	1.039 [0.322-3.353]	0.949	1.709 [0.358-8.156]	0.501	0.699 [0.110-4.447]	0.705
Group membership[e]	0.467 [0.182-1.199]	0.113	1.197 [0.375-3.819]	0.761	0.218 [0.082-0.579]	0.002 *	0.522 [0.193-1.417]	0.202
Caregiver's age	1.014 [1.005-1.023]	0.002 *	1.018 [0.990-1.046]	0.207	1.041 [0.980-1.107]	0.193	1.009 [0.992-1.027]	0.307
Flour storage duration	1.056 [1.024-1.090]	0.001 *	1.071 [0.659-1.740]	0.782	1.094 [1.017-1.176]	0.015 *	1.164 [0.871-1.556]	0.304
Cooking duration	1.215 [0.941-1.568]	0.135	1.480 [1.064-2.059]	0.020 *	1.009 [0.660-1.540]	0.968	1.684 [1.033-2.746]	0.036 *
Breastfeeding[f]	1.249 [0.807-1.935]	0.319	2.017 [0.659-6.171]	0.219	0.927 [0.3738-2.301]	0.871	1.356 [0.584-3.150]	0.478
Consumption of leftover food[g]	0.976 [0.890-1.071]	0.613	0.961 [0.839-1.100]	0.560	0.815 [0.689-0.963]	0.017 *	0.892 [0.752-1.058]	0.188
Caregiver hand washing after using latrine[h]	1.178 [0.412-3.373]	0.760	4.191 [1.149-15.290]	0.030 *	0.688 [0.304-1.556]	0.369	3.375 [0.333-34.204]	0.303

Diarrhoea in the last 14 days[i]	0.954 [0.928-0.980]	0.001 *	0.957 [0.748-1.225]	0.726	0.963 [0.791-1.173]	0.711	0.974 [0.791-1.200]	0.806
Cereals storage (sacks)[j]	0.975 [0.611-1.556]	0.915	0.729 [0.243- 2.184]	0.572	1.288 [0.562-2.956]	0.550	0.718 [0.271-1.897]	0.504
Cereals storage (granary)[k]	0.796 [0.364-1.741]	0.567	0.982 [0.315-3.061]	0.975	0.480 [0.211-1.092]	0.080	1.591 [0.590-4.295]	0.359
Constant	213.184 [101.725-446.766]	0.002	180264.1 [0.605-5.37E+10]	0.06	695.042 [2.502-193040.5]	0.023	208.943 [0.939-46481.65]	0.053
Wald $chi^2(19)$	58.3		27.45		32.83		33.26	
Prob > chi^2	0.000		0.037		0.008		0.003	
Log likelihood	-130.414		-15.600		-40.078		-41.349	
Pseudo R^2	0.216		0.583		0.393		0.329	

a, 1=male, 0=female; b, 1=crop farmer, 0=others (pastoralists, agro-pastoralists, traders, casual labourers and civil servant); c, 1=at least once, 0=never; d, 1=primary and above, 0=no formal education; e, 1=yes, the parent belongs to a group, 0=no; f, 1= yes, the child is still breastfeeding, 0=No; g, 1=more than 1 day, 0=same day; h, 1=always, 0=rarely and never; i, 1=yes, 0=no; j, 1=yes, 0=no; and k, 1=yes, 0=no. Other storage methods were considered as base category for storage methods. C.I denotes confidence interval. * Shows statistical significance (p<0.05).

Appendix C. Crude odds ratio for predictors of wasting among children under five across ecological zones

**Predictors**	**Pooled (n=240)**		**Pastoral (n=55)**		**Agro-pastoral (n=96)**		**Agricultural (n=89)**	
	**Crude Odds Ratio [95% C.I]**	**P>z**	**Crude Odds Ratio [95% C.I]**	**P>z**	**Crude Odds Ratio [95% C.I]**	**P>z**	**Crude Odds Ratio [95% C.I]**	**P>z**
Age of household head	0.974 [0.945-1.003]	0.080	0.971 [0.916-1.028]	0.313	0.944 [0.893-0.997]	0.039 *	0.987 [0.957-1.018]	0.410
Number of children under five	0.837 [0.736-0.951]	0.006 *	0.712 [0.286-1.774]	0.466	0.847 [0.583-1.231]	0.384	0.665 [0.3067-1.443]	0.302
Age of child	0.949 [0.915-0.984]	0.005 *	0.989 [0.949-1.031]	0.600	0.921 [0.870-0.974]	0.004 *	0.936 [0.896-0.977]	0.003 *
Sex of the child[a]	1.632 [1.024-2.602]	0.039 *	2 [0.648-6.177]	0.228	2.121 [0.934-4.817]	0.072	1.077 [0.463-2.504]	0.863
Education level of caregiver[b]	0.973 [0.400-2.366]	0.952	1.926 [0.590-6.289]	0.278	0.342 [0.062-1.872]	0.216	2.000 [0.314-12.733]	0.463
Household expenses	0.715 [0.579-0.884]	0.002 *	0.535 [0.261-1.097]	0.088	0.666 [0.381-1.164]	0.154	0.644 [0.408-1.017]	0.059
Age at introduction of complementary food	1.020 [1.011-1.028]	0.000 *	1.140 [0.687-1.892]	0.612	1.022 [0.710-1.472]	0.905	1.010 [0.566-1.803]	0.973
Drinking water treatment[c]	2.412 [2.323-0.504]	0.000 *	2.071 [0.558-7.684]	0.276	2.391 [1.009-5.663]	0.048 *	7.968 [0.816-22.792]	0.099
Consumption of leftover food[d]	0.617 [0.493-0.773]	0.000 *	0.886 [0.284-2.765]	0.835	0.663 [0.279-1.575]	0.352	0.351 [0.081-1.518]	0.161

Breastfeeding[e]	2.674 [1.571-4.550]	0.000 *	1.238 [0.391-3.916]	0.716	3.375 [1.285-8.862]	0.014 *	3.4 [1.407-8.214]	0.007 *
Washing of the child's hand before feeding[f]	1.135 [0.779-1.655]	0.509	6.6 [0.691-18.739]	0.128	0.381 [0.110-1.322]	0.128	1.244 [0.477-3.243]	0.655
Constant	3546.552 [1578.626-7967.706]	0.000	110171.9 [1.308-9.28E+09]	0.045	30202.38 [0.243-3.75E+09]	0.085	15875.67 [2.839-8.88E+07]	0.028
Wald chi^2(19)	47.8		18.08		27.75		26.98	
Prob > chi^2	0		0.080		0.004		0.005	
Log likelihood	-137.099		-24.775		-45.136		-45.703	
Pseudo R^2	0.171		0.313		0.321		0.251	

a, 1=male, 0=female; b, 1=primary and above, 0=no formal education; c, 1=treated by boiling or tablets, 0= not treated; d, 1=more than 1 day, 0=same day; e, 1=yes, 0=no; and f, 1=always, 0=rarely and never; C.I denotes confidence interval; * shows statistical significance ($p<0.05$).
